# Practical Considerations and Applied Examples of Cross-Validation for Model Development and Evaluation in Health Care: Tutorial

**DOI:** 10.2196/49023

**Published:** 2023-12-18

**Authors:** Drew Wilimitis, Colin G Walsh

**Affiliations:** 1 Vanderbilt University Medical Center Vanderbilt University Nashville, TN United States

**Keywords:** predictive modeling, cross-validation, tutorial, model development, risk detection, clinical decision-making, electronic health care, eHealth data, health care data, data validation, artificial intelligence, AI

## Abstract

Cross-validation remains a popular means of developing and validating artificial intelligence for health care. Numerous subtypes of cross-validation exist. Although tutorials on this validation strategy have been published and some with applied examples, we present here a practical tutorial comparing multiple forms of cross-validation using a widely accessible, real-world electronic health care data set: Medical Information Mart for Intensive Care-III (MIMIC-III). This tutorial explored methods such as K-fold cross-validation and nested cross-validation, highlighting their advantages and disadvantages across 2 common predictive modeling use cases: classification (mortality) and regression (length of stay). We aimed to provide readers with reproducible notebooks and best practices for modeling with electronic health care data. We also described sets of useful recommendations as we demonstrated that nested cross-validation reduces optimistic bias but comes with additional computational challenges. This tutorial might improve the community’s understanding of these important methods while catalyzing the modeling community to apply these guides directly in their work using the published code.

## Background

By learning complex statistical relationships from historical data, predictive models enable automated and scalable risk detection and prognostication, which might inform clinical decision-making. Although relatively few have been implemented in clinical use compared with the number developed, predictive models are increasingly being deployed and tested in clinical trials. The importance of predictive modeling is on the rise, with increasing attention from regulatory bodies such as the US Food and Drug Administration. Efforts to standardize the steps in model development and validation include statements such as transparent reporting of a multivariable prediction model for individual prognosis or diagnosis and multiple published guidelines on deployment and governance [1-3]. However, the mode in a critical step in model development, the validation strategy, remains a simple “holdout” or “test-train split,” which has been shown to introduce bias, fail to generalize, and hinder clinical utility [4-6].

Broadly, validation consists of either internal validation, which should be reported alongside model development, or external validation, in which a developed model is tested in an unseen data set in a new setting [7,8]. A newer concept of “internal-external” validation has also been suggested for studies with multisite data [9]. Most published models evaluate performance metrics by splitting the available data set into an independent “holdout” or “test set,” consisting of unforeseen samples excluded from model training. Such held-out sets are often selected randomly, for example, “80% training and 20% testing,” from the data in the original model development setting. In contrast to holdout validation, cross-validation and resampling methods such as bootstrapping can be used to produce less biased estimates of the true out-of-sample performance (ie, the ability to generalize to new samples). Although cross-validation is a widely used and extensively studied statistical method, many variations of cross-validation exist with respective strengths and weaknesses, distinct use cases for model development and performance estimation that are often misapplied, and domain-specific considerations necessary for effective health care implementation [10,11].

Cross-validation surveys with practical examples, such as those involving microarray and neurologic data, have been published [12,13]. However, gaps in comprehensive tutorials including complete codesets with relevant tutorial data are less well disseminated. Tutorials that move beyond simulated data or laboratory-based samples to real-world health care data sets might add to the understanding of these important methods while catalyzing the modeling community to apply these guides directly in their work using the published code.

The intent of this tutorial is to define and compare means of cross-validation using representative, accessible data based in the well-known and well-studied Medical Information Mart for Intensive Care-III (MIMIC-III) data set [14]. All cross-validation modeling experiments and preprocessing codes will be provided through reproducible notebooks that will further guide readers through the comparisons and concepts introduced [15]. Best practices and common missteps, particularly in modeling with electronic health care data, will be emphasized.

## Overview and Major Types of Cross-Validation

The goal of supervised learning is to use a data set with known labels, *D*(*X_i_*,*Y_i_*), to produce a model 
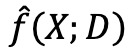
 that accurately predicts the true labels of unforeseen “test” samples *Y_i_*. 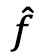 must learn robust relationships between the covariate features [X_1_... X_n_] and the outcome of interest. The model is considered a statistical estimator of *Y_i_* because the model prediction is calculated from the available training data, and *Y_i_* is a random variable with an unknown probability distribution. Given finite data samples with inherent statistical noise, the generalization or “test” error of this estimator will be imperfect. Assuming the true label to be a continuous outcome, we can decompose the mean-squared error of the learned model into 2 fundamental sources of error: *bias* and *variance*, formalized in the equation below by the first and second terms on the right hand side, respectively. The s^2^ term represents irreducible, independent, and identically distributed error terms attributed to noise in the training data set.



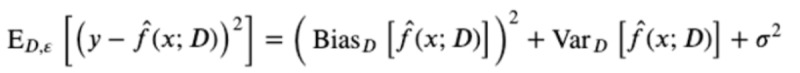



**(1)**

Understanding the tradeoff between *bias* and *variance* is necessary to develop useful predictive models in health care. Bias can be thought of as the model’s inability to discern complex statistical patterns associated with true test labels. Variance is the additional error owing to the model mistakenly interpreting random fluctuations in the training data set as a robust predictive signal. Bias can often be reduced by increasing the complexity of the model (ie, if the model is *underfit)* in hopes of uncovering deeper statistical patterns within the training data. However, the tradeoff between bias and variance then occurs, as more complex models are liable to *overfit* to random noise in the training data (thus increasing the variance). Model validation strategies such as cross-validation also have implications for the *bias-variance tradeoff*. Cross-validation generally relates to this tradeoff, as larger numbers of folds (smaller numbers of records per fold) tend toward higher variance and lower bias, whereas smaller numbers of folds tend toward higher bias and lower variance.

Before delving into the technical details and comparative advantages of specific cross-validation methods, it is imperative to emphasize that cross-validation was developed as a method to estimate the *expected* out-of-sample prediction error of a model learned from a set of training data. Machine learning developers have typically lacked access to external data sets (the gold standard that allows direct estimation of the true out-of-sample prediction error), and cross-validation offers an improvement over existing internal validation methods such as holdout validation. In contrast to parametric, model-specific methods such as Bayesian Information Criteria that rely on strict assumptions, cross-validation is nonparametric, compatible with any supervised learning algorithm, and directly estimates the primary measure of model validity—whether predictive performance generalizes to new data points. Cross-validation has become increasingly prominent for internal validation in health care given its flexibility with diverse and sophisticated learning algorithms and the advantage of using all available data for model evaluation and selection (compared with using a single holdout validation set). The use of cross-validation over holdout validation is particularly advantageous with health care data sets that are often comparatively small to moderately sized, costly to obtain, or restricted by privacy and regulatory concerns.

Cross-validation originated in the 1930s with K-fold cross-validation, the most common form of cross-validation, described in the 1960s [16,17]. In this form of cross-validation, the development data set is split into some number, k—often 5 or 10—parts, or “folds,” as described below (Multimedia Appendix 1). Several variations of this approach have since been described, each with its own advantages and disadvantages for clinical modeling.

## Considerations for Clinical Prediction and Implications for Cross-Validation

Most published predictive modeling studies focus on the classification of binary outcomes; however, fewer models have been developed to predict continuous or ordinal variables. Clinical data, especially those in secondary use, for example, electronic health records (EHRs), are also typified by (1) irregular time-sampling, (2) inconsistent repeated measures, and (3) sparsity and rarity. Missingness, noise, and anomalous outlier values are additional complicating factors associated with EHR data. Although not uniquely relevant to cross-validation, appropriate ways of handling these data issues within model development and evaluation pipelines are covered in the applied demonstrations and available code accompanying this tutorial.

As health care delivery varies naturally and widely between individuals, real-world health care data such as EHRs usually contain irregularly and inconsistently sampled measures within and across individuals. This factor has significant implications for cross-validation and is described by the differences between *subject-wise* and *record-wise* cross-validations. Subject-wise cross-validation maintains identity across splits, such that an individual’s set of events cannot exist in both training and testing simultaneously. In the record-wise cross-validation approach, data are split by event and not by individual. Record-wise cross-validation thereby increases the risk that the same individual will have events split across training and testing. A model might then achieve a spuriously high apparent performance simply by reidentifying the individual in testing based on highly similar inputs used in training.

Although the technical details and practical guidelines of subject-wise versus record-wise cross-validation are debated, the best approach depends on the specific use case, the number of records, the size of the data set, and the degree of correlation within subject records [18]. Developers should also consider the unit of modeling, that is, making a prediction for a given person versus a given health care encounter or event such as a prescription. In the former example, record-wise cross-validation might be adopted for diagnosis at a given clinical encounter, and subject-wise cross-validation would be favorable for prognosis over time [19]. In the latter examples, the training data might include multiple events per person, and the number of events will also vary across individuals. Cross-validation poses an additional potential benefit in that training and testing strategies might split individuals in such a way that models can be trained on some folds and then applied as inputs for ensembling in different folds without increasing the risk of overfitting or data leakage [20].

Many clinical outcomes subject to predictive modeling studies are rare at the health-system scale (eg, ≤1% incidence). Although rare outcomes create modeling challenges out of scope for this tutorial, they also impact cross-validation. Randomly partitioning data sets into training and test splits often produces folds with various outcome rates and even folds with no outcome instances. For binary classification problems, stratified cross-validation ensures that outcome rates are equal across folds, and it is recommended for classification problems (and should be considered necessary for highly imbalanced classes) [20].

## Major Steps in Using Cross-Validation

Dividing steps into development steps and validation steps eases interpretation. Development steps include data cleaning and preprocessing—a time-consuming but critical task given noisy and often invalid health care data from real-world sources–feature selection, classifier selection, hyperparameter tuning, and model refitting (Textbox 1). For brevity and scope, classifier selection will not be covered in detail but is emphasized as an important step in any predictive modeling pipeline. As classifiers, broadly parametric or nonparametric, require different assumptions and themselves pose disparate advantages and disadvantages, it has become standard to test multiple classifiers in the same modeling study. Moreover, ensembles of these classifiers are increasingly developed given the rise in complexity, depth, and breadth of real-world health care data sets in the literature.

Model performance metrics inform validation steps and, when properly contextualized in the clinical use case, suggest key metrics on which to either optimize or evaluate model performance. A detailed discussion of model performance metrics remains outside the scope of this tutorial and has been covered in depth elsewhere. In brief, prediction models must be evaluated in terms of discrimination (ie, the ability to predict higher probabilities for individuals with the outcome) and calibration (a measure of similarity between predicted probabilities and the observed risk) [21]. Two methods to evaluate discrimination via the area under the receiver operator characteristic curve (AUROC) and the area under the precision-recall curve (AUPR) from cross-validation include (1) pooling: averaging test-fold results at each point on the receiver operating characteristic or precision-recall curve and (2) averaging: reporting the average AUROC and AUPR over each test-fold metric. Calibration can also be assessed analogously using metrics such as the Brier score, calibration slope, and intercept. We highlight methods that can be used for calibrating predictions along with cross-validation in a provided Jupyter notebook. Clinical usefulness based in decision analysis is the third major area of evaluation [22]. Usefulness bridges model performance to utility, for example, showing how a model might reduce cost or increase the measurement of quality of life.

In addition to cross-validation, bootstrapping is another resampling-based method used to provide more accurate estimates of the model generalization performance than holdout validation. Bootstrapping involves randomly sampling with replacement from the entire data set to generate a training set that will not include all original samples. A model is then fitted on the bootstrap training set and evaluated on a test set comprising the remaining unselected observations. This process is repeated several times, where the number of iterations is typically referred to as the number of bootstraps, and a CI is generated from the collection of out-of-sample (sometimes called “out of bag”) performance metrics. Traditional bootstrapping is referred to as out-of-bag bootstrapping, whereas further improvements include the “0.632” and “0.632+” methods that apply additional forms of bias adjustment [23,24]. More advanced resampling methods include bootstrap-based cross-validation, Monte Carlo holdout validation (or repeated holdout sampling), and repeated nested cross-validation [25,26].

**List of steps required for cross-validation.**

1. *Data cleaning* (outside the loop)—Basic manipulation and feature engineering (converting data types, one-hot label encoding, etc) can and should be completed on the entire data set before beginning cross-validation.
2. *Feature scaling and imputation* (within the loop)—Imputation and feature scaling based on the other values in the data set (such as standardization via mean-centering or normalization) need to be completed only on the training set—which we call “within the loop” as a reference to use with nested cross-validation. This is necessary to reduce data leakage that can be caused if values in the test set are used to impute or scale the values in the training set.
3. *Feature selection* (within the loop)—To reduce overfitting and the detection of spurious correlations between the outcome and independent variables, feature selection should be completed only on the training fold (“inner loop” of nested cross-validation) and then applied and evaluated on the test fold.
4. *Model selection* (within the loop)—To mitigate optimistic bias, the comparison of different modeling algorithms (eg, random forest vs logistic regression) should also be completed separately from model evaluation.
5. *Model selection* (within the loop)—Optimizing hyperparameters can also be done simultaneously with classifier selection and should be used when identifying the best modeling algorithm.
6. *Evaluation*—Evaluation by way of “averaging” or “pooling” should be completed separately from using cross-validation for model selection to reduce optimism resulting from overfitting parameters to the data set used for evaluation.
7. *Model refitting*—To produce a final model trained with all the available data, one should learn the ideal feature selection and model selection parameters from cross-validation and then train the selected algorithm with these parameters using the entire data set. The model can then be ported outside the development setting to use for external validation or within production-grade systems.

## Case Study in Cross-Validation: In-Hospital Mortality and Length of Stay Prediction

MIMIC-III represents a well-known deidentified data set based in intensive care at Beth Israel Deaconness for approximately 40,000 patients who received care from 2001 to 2012 [14]. It has been studied extensively, given its relative accessibility compared with most health care modeling studies using EHRs, in which privacy or challenges in at-scale deidentification prohibit data sharing with publication [27].

To demonstrate the key concepts in cross-validation, we selected 2 exemplary problems that typify predictive modeling studies: classification and regression. For this case study, in-hospital mortality prediction will represent the former, whereas length of stay prediction will represent the latter. The models will be developed and validated using multiple forms of cross-validation, including K-fold, stratified, repeated, repeated stratified, and nested cross-validation. We also applied bootstrap methods to generate CIs for estimated model performance.

From patient visit records, we derived time-invariant features such as age, sex, and race, along with binary features indicating the presence of prior diagnoses using 25 higher-order categories of International Classification of Diseases codes grouped into Clinical Classifications Software codes. In-hospital mortality was defined as a binary classification problem, where 1 indicated that mortality occurred at any point during the hospital visit and 0 otherwise. Length of stay was defined in days and used as a continuous outcome for a separate regression prediction problem.

Preprocessing included imputation of continuous features using the median, setting outlier age values to a maximum of 110 years, and standardization of all numerical features. We also applied a feature selection routine to select only the top 10 features available for in-hospital mortality prediction and either the top 30 or 50 features (where the number of features was included as a hyperparameter) for length of stay prediction. Finally, in-hospital mortality prediction was classified using logistic regression and grid search over hyperparameters including least absolute shrinkage and selection operator (L1), Ridge (L2), and no penalization and a range of regularization values. Length of stay prediction was performed using random forest regression and grid search over hyperparameters including the number of estimators and maximum tree depth.

**Figure 1:**
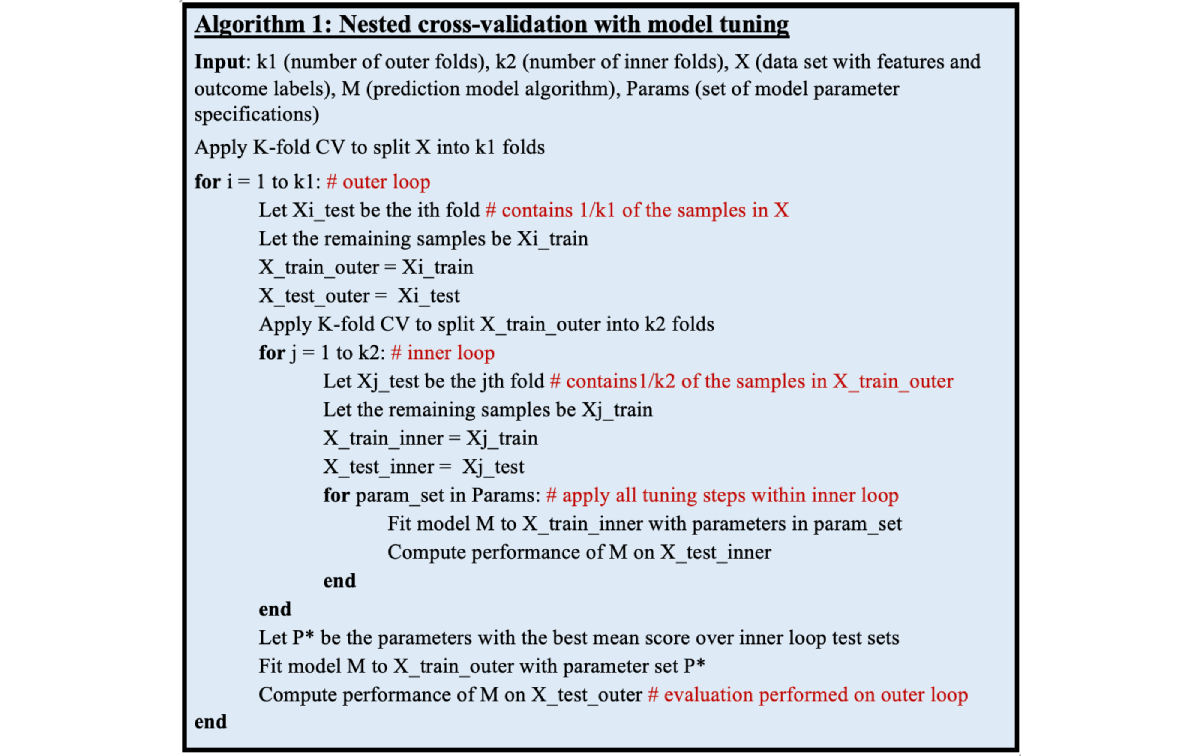
Pseudocode for nested cross-validation algorithm with model tuning.

In accordance with the best practices outlined for cross-validation and model selection in Figure 1, we implemented a nested cross-validation approach that performed all hyperparameter tuning and model selection steps within the “inner” cross-validation loop. Theoretically, this should mitigate the source of optimistic bias introduced when cross-validation is used to tune model parameters on the same data used for model performance evaluation (ie, observed performance can be spuriously high owing to randomness in the data and the learning algorithm) [28,29]. This source of bias in the estimated model performance can be considered as a type of overfitting in the model selection procedure [30].

To empirically evaluate whether nested cross-validation produces more accurate performance estimates than nonnested cross-validation, we compared the nested cross-validation with nonnested cross-validation used simultaneously for model selection and model evaluation. For nonnested cross-validation methods, we evaluated the performance of each set of model tuning configurations (eg, models trained with varied hyperparameters) on the test fold at each cross-validation split. After repeating this procedure for each split within the given cross-validation method, we computed the average performance over all test folds for each model tuning configuration. The model parameters with the best average performance over the cross-validation test folds were then selected. The performance of this optimal set of hyperparameters was then reported as an estimate of true out-of-sample performance.

**Figure 2:**
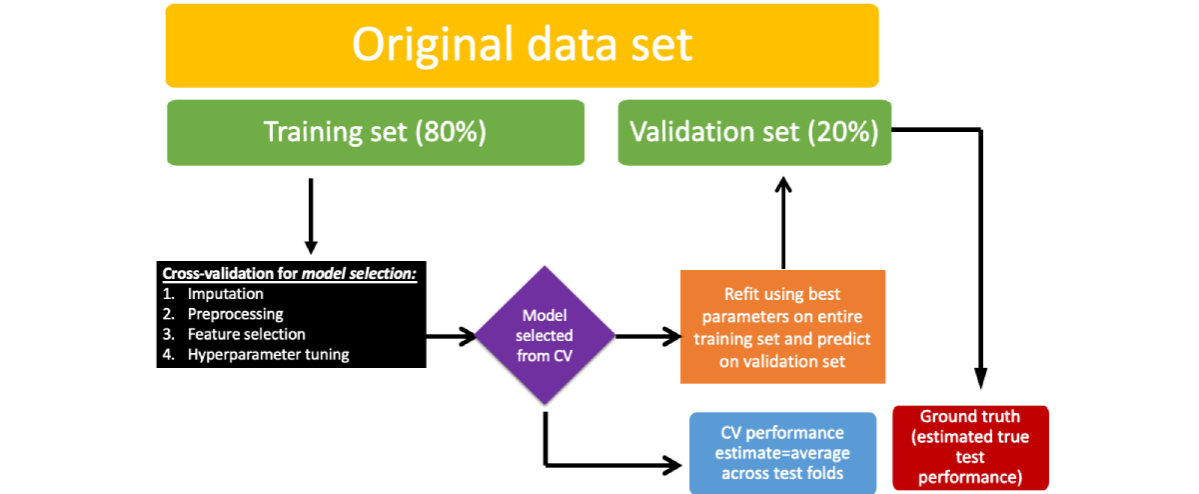
Diagram of the methodology for cross-validation (CV) optimism error estimation experiment.

To demonstrate the optimism that can result from improperly applied model validation strategies (ie, simultaneously applying nonnested cross-validation methods for both model selection and model evaluation), we evaluated the accuracy of the estimated true test performance when using various cross-validation methods. We performed this by randomly splitting the data set into an 80% (32,897/41,121) sample used for cross-validation and a 20% (8224/41,121) withheld validation sample. We used a holdout setup to simulate ground truth in the absence of a naturally bounded holdout (eg, by site or clinical setting) in the MIMIC data. We then compared the best model performance reported from cross-validation with the performance of that model when predicting on the held-out validation set (Figure 2).

Performance measures will include discrimination metrics such as the AUROC and AUPR. For length of stay regression modeling, we adopted mean absolute error and median absolute error as the primary performance metrics. Computational time, a pragmatic concern affecting many modeling experiments, will also be compared across cross-validation methods for both prediction outcomes.

## Case Study Results

### Cohort Description

After applying the exclusion criteria, our cohort included 41,121 hospital visits that comprised 71.63% (29,457/41,121) of White patients and 55.92% (22,996/41,121) of male patients. Mortality was observed for 4320 patient visits (4320/41,121, 10.51% of the total cohort). The length of stay (days) did not vary across different demographic groups, whereas the mean age of patients with in-hospital mortality (68.7, SD 15.0 y) was greater than those without mortality events (61.6, SD 16.7 y). Among visits in which mortality occurred, the most common primary admission reasons were brain hemorrhage (256/4320, 5.93%) and sepsis (201/4320, 4.65%). Cardiac arrest and hypoxia showed the highest length of stay, with mean values of 5.3 (SD 5.8) and 5.1 (SD 5.6) days, respectively. For prior Clinical Classifications Software diagnostic history, patients with pneumonia, respiratory failure, arrest, and insufficiency, and shock had a mean length of stay of approximately 7 days (SD 8.6, 8.2, and 8.2, respectively). The proportion of in-hospital mortality events was highest for patients diagnosed with respiratory failure, arrest, and insufficiency, fluid and metabolic disorders, and renal failure (Table 1).

**Table 1:** Medical Information Mart for Intensive Care-III patient cohort summary by mortality and average length of stay (N=41,121).

			In-hospital mortality					Length of stay (days)		
			0		1		Values, mean (SD)			Values, median (IQR)
**Sex, n (%)**										
	Male	20,717 (56.29)		2279 (52.75)		3.64 (5.26)			1.99 (1.16-3.74)	
	Female	16,084 (43.71)		2041 (47.25)		3.63 (5.17)			2.05 (1.17-3.78)	
**Race, n (%)**										
	Asian	847 (2.3)		116 (2.69)		3.61 (5.36)			2.0 (1.19-3.54)	
	Black	3663 (9.95)		286 (6.62)		3.42 (5.01)			1.99 (1.16-3.41)	
	Hispanic	1369 (3.72)		82 (1.9)		3.24 (4.21)			1.89 (1.12-3.36)	
	White	26,421 (71.79)		3036 (70.28)		3.61 (5.21)			2.01 (1.16-3.72)	
	Other or unknown	4501 (12.23)		800 (18.52)		4.04 (5.61)			2.13 (1.2-4.14)	
**Age**										
	Not applicable, %	1739 (4.73)		447 (10.35)		—^a^			—	
	Values, mean (SD)	61.63 (16.71)		68.67 (14.99)		—			—	
**Height**										
	Values, mean (SD)	169.01 (13.39)		167.46 (13.22)		—			—	
	Not applicable, %	28,383 (77.13)		3420 (79.17)		—			—	
**Weight**										
	Values, mean (SD)	82.2 (24.14)		77.39 (23.4)		—			—	
	Not applicable, %	6541 (17.77)		709 (16.41)		—			—	
**Admission reason, n (%)**										
	Brain hemorrhage	517 (1.4)		256 (5.93)		4.01 (5.13)			1.93 (1.05-4.74)	
	Cardiac arrest	105 (0.29)		114 (2.64)		5.27 (5.75)			3.8 (1.7-6.81)	
	Sepsis	747 (2.03)		201 (4.65)		4.71 (7.19)			2.49 (1.5-4.7)	
	Respiratory distress	96 (0.26)		25 (0.58)		4.28 (4.45)			2.62 (1.41-5.08)	
	Liver failure	107 (0.29)		39 (0.9)		4.76 (5.38)			2.78 (1.63-5.8)	
	Hypoxia	81 (0.22)		21 (0.49)		5.07 (5.61)			2.87 (1.65-5.65)	
	Cerebrovascular accident	54 (0.15)		18 (0.42)		3.26 (4.29)			1.99 (1.06-3.34)	
**CCS^b^ diagnoses: cardiovascular, n (%)**										
	Acute myocardial infarction	3708 (10.08)		559 (12.94)		3.83 (4.82)			2.25 (1.3-4.2)	
	Coronary atherosclerosis and other heart disease	12,193 (33.13)		1091 (25.25)		3.22 (4.07)			2.03 (1.17-3.45)	
	Cardiac dysrhythmias	11,486 (31.21)		1724 (39.91)		4.26 (5.92)			2.3 (1.29-4.44)	
	Essential hypertension	15,685 (42.62)		1604 (37.13)		3.31 (4.39)			1.99 (1.16-3.43)	
	Hypertension with complications and secondary hypertension	4738 (12.87)		649 (15.02)		3.77 (5.33)			2.11 (1.22-3.98)	
	Congestive heart failure and nonhypertensive	9482 (25.77)		1482 (34.31)		4.45 (6.01)			2.54 (1.4-4.85)	
	Conduction disorders	2603 (7.07)		335 (7.75)		3.58 (4.60)			2.16 (1.24-4.02)	
**CCS diagnoses: diabetes and metabolic, n (%)**										
	Fluid and electrolyte disorders	9195 (24.99)		1823 (42.2)		4.51 (5.91)			2.45 (1.38-4.92)	
	Disorders of lipid metabolism	11,162 (30.33)		812 (18.8)		2.96 (3.77)			1.89 (1.13-3.14)	
	Diabetes mellitus without complication	7044 (19.14)		872 (20.19)		3.71 (5.12)			2.08 (1.19-3.95)	
	Diabetes mellitus with complications	3597 (9.77)		288 (6.67)		3.46 (4.96)			2.07 (1.21-3.44)	
**CCS diagnoses: infectious disease, n (%)**										
	Septicemia (except in labor)	4332 (11.77)		1497 (34.65)		6.48 (8.53)			3.18 (1.78-7.54)	
	Pneumonia (except that caused by tuberculosis or sexually transmitted disease)	4594 (12.48)		1094 (25.32)		7.15 (8.60)			3.89 (1.89-8.99)	
**CCS diagnoses: kidney and gastrointestinal and liver, n (%)**										
	Acute and unspecified renal failure	6911 (18.78)		1855 (42.94)		5.26 (7.22)			2.77 (1.56-5.75)	
	Other liver diseases	2847 (7.74)		804 (18.61)		4.98 (6.88)			2.57 (1.44-5.14)	
	Gastrointestinal hemorrhage	2567 (6.98)		430 (9.95)		4.06 (6.14)			2.09 (1.25-4.04)	
	Chronic kidney disease	4780 (12.99)		657 (15.21)		3.63 (4.93)			2.12 (1.22-3.91)	
**CCS diagnoses: respiratory, n (%)**										
	Respiratory failure, insufficiency, and arrest (adult)	5361 (14.57)		2042 (47.27)		7.30 (8.21)			4.37 (2.1-9.31)	
	Other upper respiratory disease	1514 (4.11)		143 (3.31)		5.46 (7.31)			2.9 (1.47-6.17)	
	Other lower respiratory disease	1892 (5.14)		245 (5.67)		4.20 (5.73)			2.25 (1.28-4.56)	
	Chronic obstructive pulmonary disease and bronchiectasis	4642 (12.61)		684 (15.83)		4.24 (5.86)			2.26 (1.27-4.61)	
	Pleurisy, pneumothorax, and pulmonary collapse	3139 (8.53)		454 (10.51)		5.78 (7.7)			3.03 (1.7-6.3)	
**CCS diagnoses: stroke, n (%)**										
	Acute cerebrovascular disease	2248 (6.11)		798 (18.47)		5.11 (6.51)			2.62 (1.35-6.16)	
**CCS diagnoses: surgical complications or shock, n (%)**										
	Complications of surgical procedures or medical care	7823 (21.26)		719 (16.64)		5.18 (7.33)			2.61 (1.36-5.24)	
	Shock	2017 (5.48)		1173 (27.15)		6.85 (8.15)			3.89 (1.99-8.4)	

^a^Stratified summary statistics were indeterminable for continuous demographic variables.

^b^CCS: Clinical Classifications Software.

### In-Hospital Mortality Prediction

**Figure 3:**
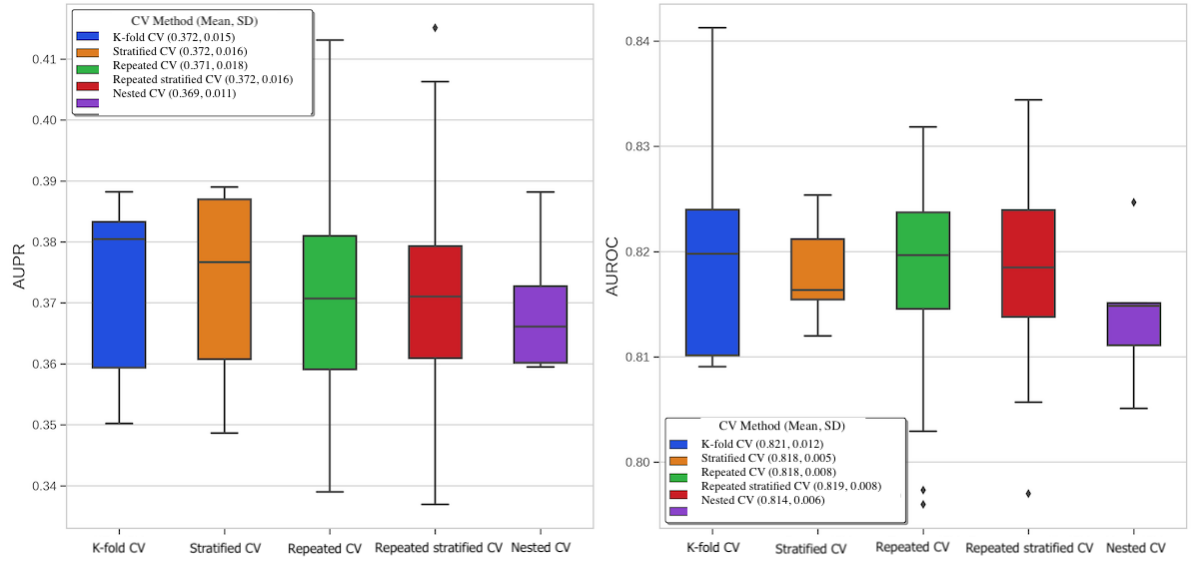
Discrimination for in-hospital mortality prediction by cross-validation (CV) method (with 5 folds used for each method). AUPR: area under the precision-recall curve; AUROC: area under the receiver operator characteristic curve.

In a comparison of cross-validation approaches for in-hospital mortality prediction (including all model selection steps and setting the number of folds to 5 for each method), stratified K-fold cross-validation performed approximately the same as regular K-fold cross-validation. Repeated methods of cross-validation performed marginally worse than the simple methods of cross-validation, whereas wider spreads of performance metrics were observed for repeated methods. Nested cross-validation performed slightly worse than both repeated and simpler methods, with a mean AUPR value of 0.369 (compared with 0.371-0.372) and an AUROC value of 0.814 (compared with 0.818-0.821). Across all cross-validation methods, discrimination was moderate to strong for in-hospital mortality prediction, likely owing to the case prevalence of 10.51% (4320/41,121) and the availability of relevant predictive features (demographics, diagnostic history, and admission criteria; Figure 3).

**Figure 4:**
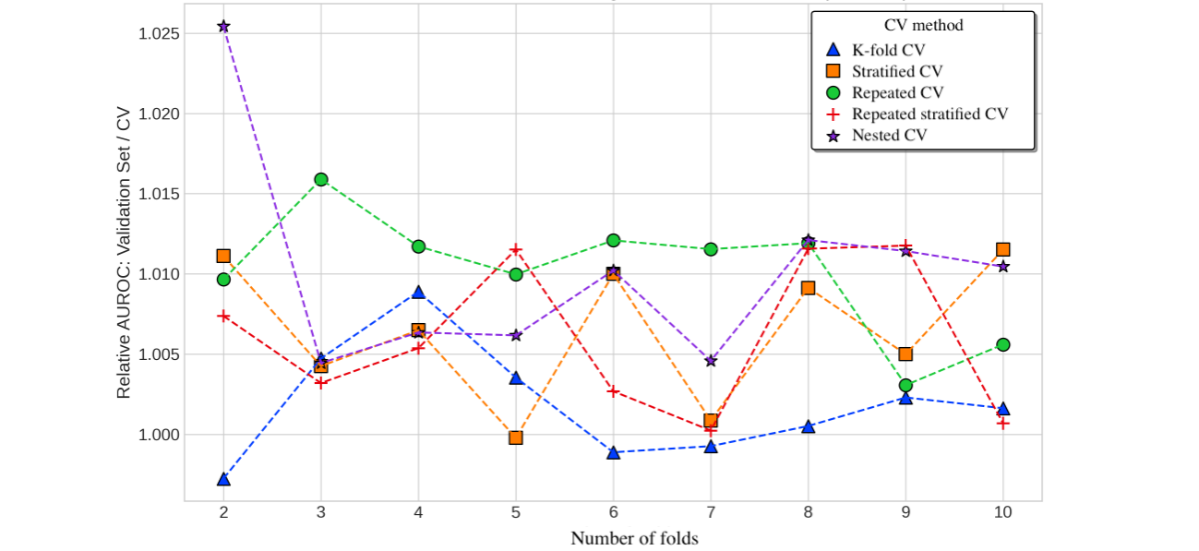
Cross-validation (CV) estimates versus validation set performance (AUROC) for in-hospital mortality by number of folds. AUROC: area under the receiver operator characteristic curve.

**Figure 5:**
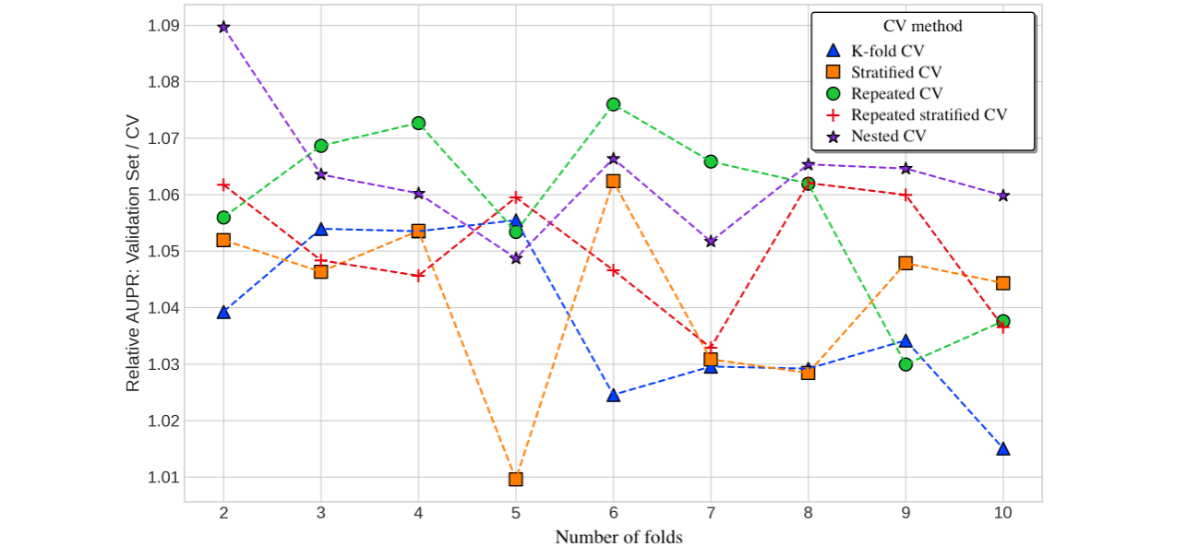
Cross-validation (CV) estimates versus validation set performance (AUPR) for in-hospital mortality by number of folds. AUPR: area under the precision-recall curve.

To assess whether nested cross-validation mitigates overfitting and optimistic bias compared with nonnested methods, we compared the performance estimate given by cross-validation (the average over test folds) with the performance of a model trained on the entire data set with the optimal hyperparameters from cross-validation. We used this refitted model and made predictions on an entirely withheld validation set (comprising 8224/41,121, 20% of the total data set vs 32,897/41,121, 80% used for cross-validation with model selection). The y-axes in Figures 4 and 5 show that the cross-validation estimate had a slight pessimistic bias, as the ratio of validation set performance divided by the cross-validation estimate was >1. Cross-validation estimates slightly underestimated out-of-sample performance. The discrepancy between the cross-validation estimates and validation set performance was greatest for lower numbers of folds. We only observed marginal differences in the degree of pessimistic bias across the cross-validation methods, although AUPR estimates had a greater bias than AUROC. Estimates from nested cross-validation and repeated K-fold cross-validation were the most pessimistically biased (approximately 1%-2% for AUROC and 5%-9% for AUPR), whereas K-fold cross-validation was the least pessimistically biased (Figures 4 and 5).

Over 100 bootstrap iterations, the 0.632 bootstrap method had a mean AUPR of 0.368 (95% CI 0.351-0.382) and a mean AUROC of 0.819 (95% CI 0.813-0.825). The out-of-bag bootstrap method had a mean AUPR of 0.367 (95% CI 0.346-0.390) and a mean AUROC of 0.818 (95% CI 0.796-0.828).

**Figure 6:**
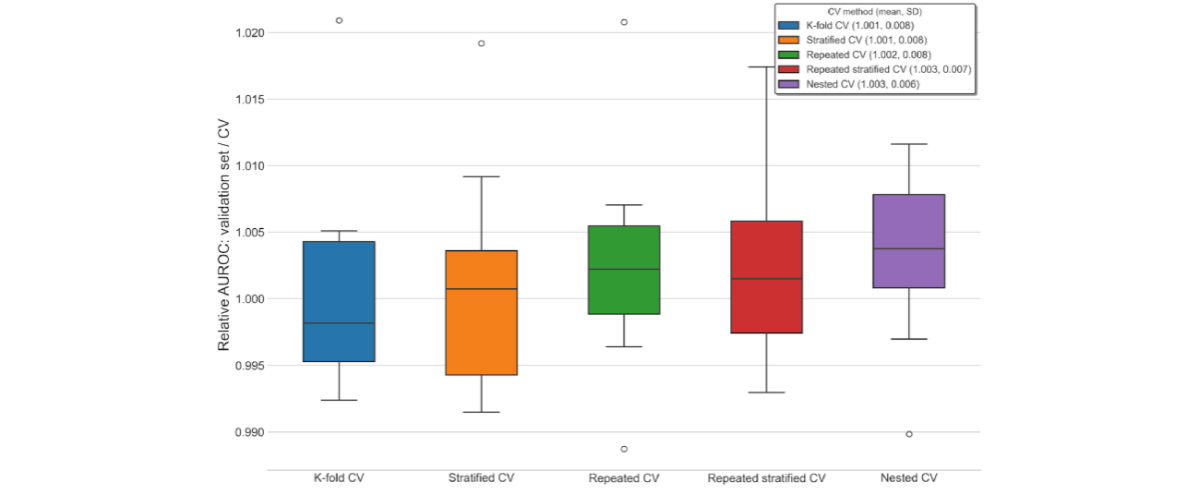
Cross-validation (CV) estimates versus validation set performance (AUROC) for in-hospital mortality over repeated 5-fold trials. AUROC: area under the receiver operator characteristic curve.

**Figure 7:**
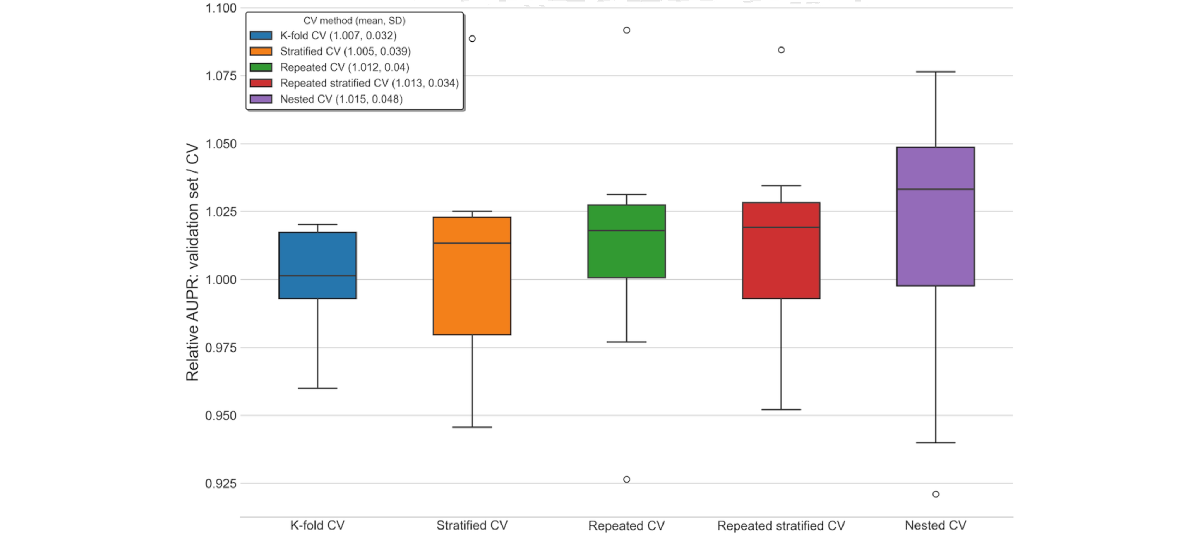
Cross-validation (CV) estimates versus validation set performance (AUPR) for in-hospital mortality over repeated 5-fold trials. AUPR: area under the precision-recall curve.

We also repeated the optimism estimation experiment using cross-validation methods (each specified with 5 folds) applied to 10 randomly sampled validation sets for a more robust estimation of the model performance bias. Nested cross-validation showed a marginally greater pessimistic bias than nonnested cross-validation methods for both AUROC and AUPR. Over the 10 randomly sampled validation sets, outlier values for the relative error of the cross-validation estimate ranged from 8% optimistic bias (AUPR for nested cross-validation) to 10% pessimistic bias (AUPR for all nonnested methods; Figures 6 and 7).

**Figure 8:**
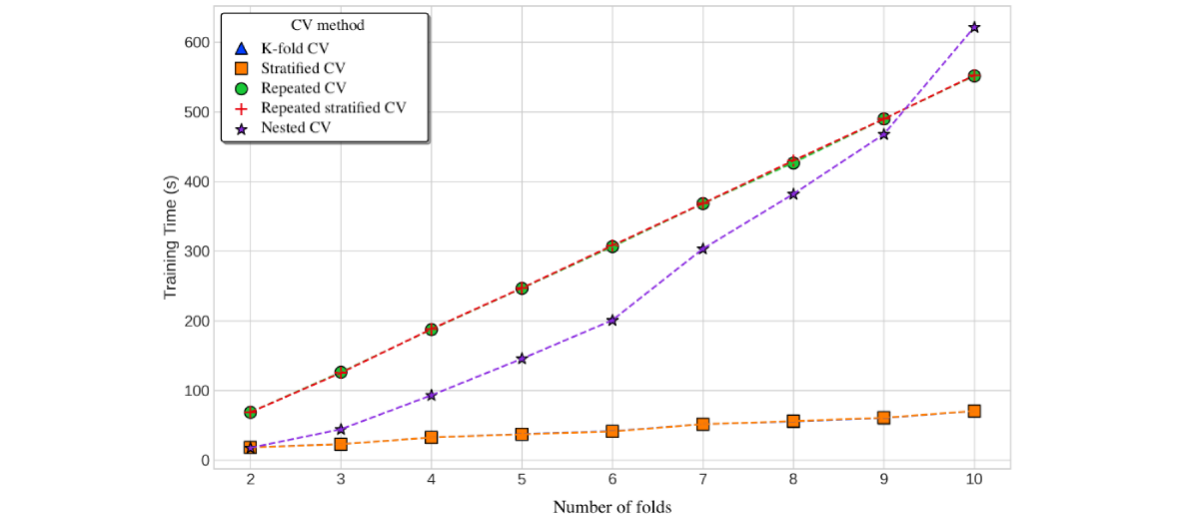
Computational time required by cross-validation (CV) method and number of folds for in-hospital mortality prediction. CV: cross-validation.

In addition to modest performance differences, tendencies toward increased computational time were observed with a more sophisticated schema, for example, nested cross-validation. Although the overall training time differences were inconsequential for this data set, the computational time of the nested cross-validation increased quadratically with the number of folds (O(k^2^)). In comparison, the computational time required for the repeated cross-validation methods increased linearly (O(k)) and simple cross-validation methods required nearly constant time across varying number of folds (O(c)) (Figure 8).

### Length of Stay Prediction

**Figure 9:**
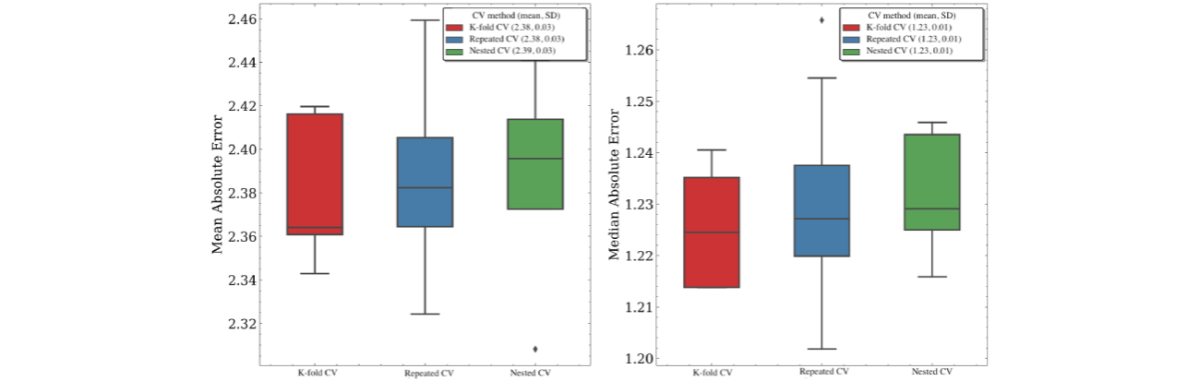
Regression metrics by cross-validation (CV) method for length of stay prediction (with 5 folds used for each method).

With length of stay prediction defined as a regression problem, we compared the test-fold performance metrics across various cross-validation methods (with each method using 5 folds). We were unable to include stratified cross-validation, which is only applicable to classification problems wherein the case prevalence can be made equivalent across different training and test folds. Similar to in-hospital mortality prediction, we observed equivalent or marginally worse performance for nested cross-validation compared with nonnested methods (with average mean absolute errors of 2.39 vs 2.38 for nested vs nonnested methods, and average median absolute errors of 1.23 for all methods). The mean absolute error was nearly twice that of the median absolute error, which suggests that higher outlier values for length of stay increased the mean prediction error in this regression problem (Figure 9).

The 0.632 bootstrap method had an average mean absolute error of 2.01 (95% CI 1.98-2.04) and an average median absolute error of 1.05 (95% CI 1.03-1.07). The out-of-bag bootstrap method had an average mean absolute error of 2.84 (95% CI 2.79-2.90]) and an average median absolute error of 1.53 (95% CI 1.49-1.55).

**Figure 10:**
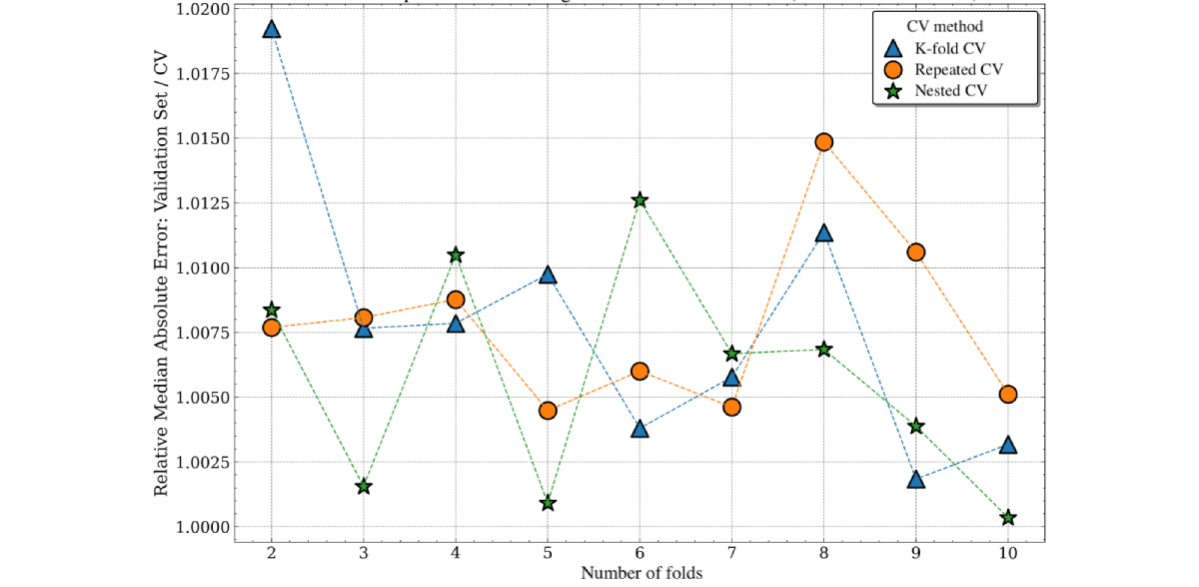
Cross-validation (CV) estimates versus validation set performance (Median Absolute Error) by number of folds for length of stay prediction.

For median absolute error, all cross-validation methods showed a slight pessimistic bias (because the validation set performance was slightly greater than the estimated performance from cross-validation). There were few disparities between the accuracy of the performance estimates for varying numbers of folds or different cross-validation methods. The pessimistic bias was greatest for the K-fold cross-validation with 2 folds (approximately 2%). Nested cross-validation produced the least biased estimates overall, although the bias from the nonnested methods remained <1% (Figure 10).

**Figure 11:**
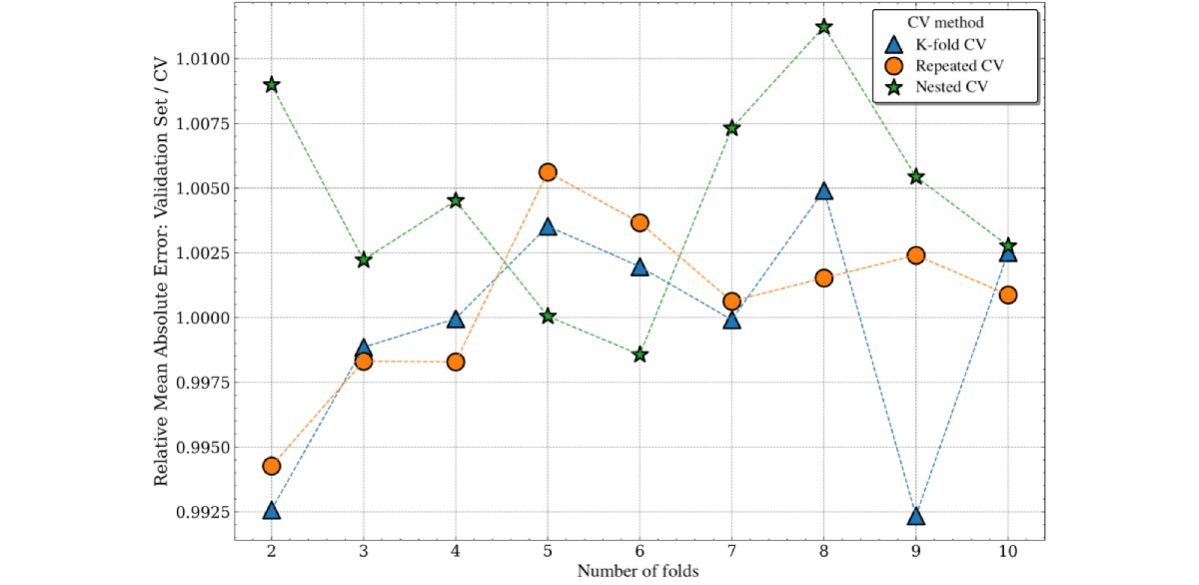
Cross-validation (CV) estimates versus validation set performance (Mean Absolute Error) by number of folds for length of stay prediction.

Repeated and single K-fold cross-validation estimates of the mean absolute error were slightly optimistically biased, whereas nested cross-validation was pessimistically biased across all folds. As the number of folds increased, the nonnested methods were generally less biased. Nested and nonnested cross-validation estimates of the mean absolute error were approximately unbiased, with a range between 1% pessimistic bias (nested cross-validation) and 1% optimistic bias (K-fold cross-validation; Figure 11).

**Figure 12:**
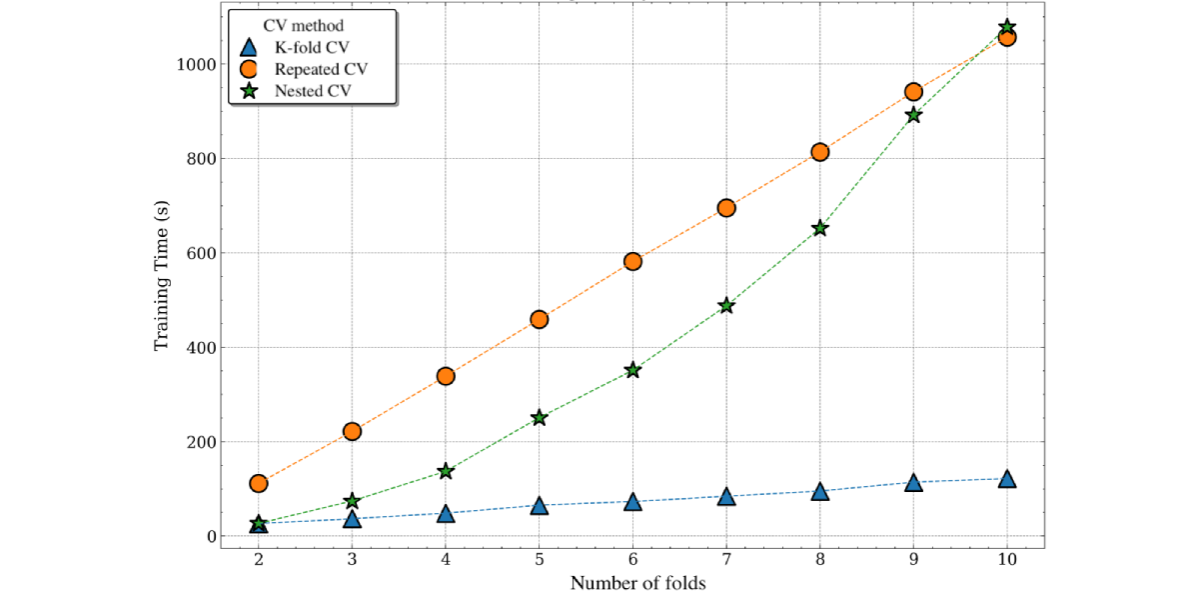
Computational time required by cross-validation (CV) method and number of folds for length of stay prediction. CV: cross-validation.

Consistent with the classification problem, we also observed a quadratic relationship between the computational time required for nested cross-validation and the number of folds. K-fold cross-validation showed linear time complexity, with repeated K-fold cross-validation increasing linearly with the additional multiplicative factor from the number of repeats. Owing to the increased training time required for ensemble models such as random forest, the absolute time required for cross-validation methods was much higher for length of stay prediction than for in-hospital mortality (Figure 12).

Finally, we tested record-wise versus subject-wise cross-validation and found negligible differences in the accuracy of the model performance estimates. We suspect that this may have resulted from the relatively few repeated hospital visits (records or observations in our data set) associated with each unique subject or the minimal correlation between the feature values of identical records across different hospital visits (ie, differences in reasons for hospital admission may have been diverse within a subject’s set of visit records).

## Recommendations, Common Missteps, and Best Practices

This tutorial described and compared multiple forms of cross-validation. Cross-validation generally results in reduced bias compared with holdout testing and poses the clear advantage of training and testing on all available data [6]. A more sophisticated schema of model validation involves bootstrapping methods (and even involving bootstrap-based cross-validation or repeated nested cross-validation). However, the modest computational time and the acceptable biased estimates of true test error that we observed suggest that the conventional cross-validation methods should remain the first line for real-world health care modeling. Although K-fold cross-validation remains the most common, other types of cross-validation pose advantages and disadvantages worth considering for each use case (Multimedia Appendix 1). Case studies using readily accessible and well-studied EHR data, MIMIC-III, showed slight performance differences in terms of cross-validation performance and optimistic bias for more computationally intensive forms of cross-validation such as nested cross-validation. Although our results should not dictate whether nested cross-validation is used across the variety of prediction problems and clinical data sets, the reduction in optimistic bias with nested cross-validation does not outweigh the additional challenges of implementing nested cross-validation and the added computational time it requires.

Common missteps detract from the potential for cross-validation in diverse modeling scenarios. For example, model development might be more complex across iterations, and separating development (feature selection, hyperparameter tuning, and classifier selection) from model validation remains paramount. When using cross-validation for model development within the same process as model validation, it is often recommended to use a nested cross-validation approach where preprocessing, feature selection, and hyperparameter tuning are conducted entirely independent of the “outer fold” that is used independently for validation.

However, as our results illustrate, and prior studies have covered both empirically and theoretically, the degree of optimistic bias from nonnested cross-validation methods varies with the number of features, the size of the data set, the extent of hyperparameter tuning and feature selection, and the nature of the clinical outcome. In this case study, we had a relatively large sample size relative to the number of original features included (referred to as n>p). When performing hyperparameter tuning and other model selection steps within cross-validation, the difference in optimistic bias observed between nested and nonnested cross-validation methods should have been mitigated [31]. Furthermore, our range of hyperparameters and the subsequent number of possible model tuning configurations was relatively smaller than a developer would typically use when wanting to optimize performance. This also contributed to a relatively lower reduction in optimistic bias when using the theoretically validated approach to reduce optimistic bias resulting from model tuning in cross-validation (nested methods) [12,30,32].

Although it is impossible to recommend a single cross-validation approach that will be appropriate for all modeling scenarios, we encourage developers to use nested cross-validation methods in cases with higher dimensional feature spaces relative to the sample size, higher numbers of algorithms and parameters being tested, and problems in which the increase in computational time required from nested cross-validation remains within feasible bounds (as we observed in both modeling problems in our case study). As emphasized throughout our discussion of nested cross-validation, this approach also offers the simplicity of performing both model selection and tuning and model evaluation within the same procedure, allowing developers to disregard concerns about or additional evaluations needed to mitigate the bias introduced when using nonnested methods for model selection (which should be used by default to optimize model performance) and model evaluation. Although we hope to contribute further empirical evidence on the comparative bias between various cross-validation methods, we emphasize that this work is a tutorial meant to demonstrate the use of various approaches that developers can use for their specific use cases.

In routine health care, EHRs include repeated, irregular samples (records or health care encounters) across records (patients). Although we observed negligible differences in performance between subject-wise and record-wise cross-validation in this case study, the use case for predictive modeling should determine the choice between subject-wise and record-wise sampling. For example, a cohort study of encounters in an emergency department to predict admissions for pneumonia might include data sets with multiple encounters per person, some with a single encounter and others with multiple encounters. Record-wise splitting might permit encounters for the same individual to be present in both training and testing sets, even if the outcomes of each of those encounters with respect to the prediction target might differ. The tendency in health care data for correlation and, specifically, autocorrelation would also introduce undue bias in this scenario.

A fundamental misconception about cross-validation is that it necessarily “returns” a model that can then be used for production deployment or external validation [33]. Rather, cross-validation is more appropriately considered a *learning procedure*, which allows a developer to fine-tune the parameters involved in model development and estimate model performance on out-of-sample data (internal validation). Once model selection via cross-validation has produced the best selected features, hyperparameters, and modeling algorithm, it is necessary to retrain a “final model” using the entire available data set with these optimized specifications.

We hope to address the current limitations of machine learning evaluation and development that might hinder the translation and reproducibility of predictive models in health care. With respect to their specific clinical implications, we provided greater conceptual understanding of cross-validation as both a model evaluation and model development method, outlined the respective strengths and weaknesses of common cross-validation methods, specified the technical steps involved when using cross-validation with model tuning and selection, demonstrated cross-validation in a real-world case study, and offered further empirical evidence on the performance and computational time of cross-validation methods. Practically, we refer readers to our open-source code repository with reproducible Jupyter notebooks and Python code, implementing all the statistical analyses and experiments of this tutorial. Therefore, developers will have access to cross-validation examples with real-world health care data and software functionality that can aid developers with various clinical machine learning problems.

## Appendix

Multimedia Appendix 1 Major types of cross-validation with broad advantages and disadvantages.

## Abbreviations

AUPR: area under the precision-recall curve
AUROC: area under the receiver operator characteristic curve
EHR: electronic health record
MIMIC-III: Medical Information Mart for Intensive Care-III
