# Supplementary material for: Practical Considerations and Applied Examples of Cross-Validation for Model Development and Evaluation in Health Care: Tutorial
Source: JMIR AI. 2023 Dec 18;2:e49023. doi: 10.2196/49023 (PMC11041453; doi:10.2196/49023)
Supplement: Multimedia Appendix 1 [file ai_v2i1e49023_app1.docx]

| Method | Description | Advantages | Disadvantages |
| --- | --- | --- | --- |
| Holdout  validation | - Involves a single split of the data set into train and test sets — typically partitioning 80% of the data for model training and the remaining 20% for model evaluation. - Estimated generalization performance is reported as the performance of the model (fit to the training set) evaluated on the test set. | - Very intuitive approach —enticing for newer model developers applying machine learning within traditionally non-quantitative fields (as suggested by the over-reliance on holdout validation within healthcare modeling). - Less computational time required than cross-validation or resampling methods. - In theory, holdout validation provides an unbiased estimate of true test error when applied to very large or near infinite datasets because the bias generally decreases as the size of the training set increases according to the BVT^a^ (although this attribute is almost always irrelevant with real-world data). | - More optimistically biased estimate of true test error (i.e., performance reported by holdout validation is typically better than the true out of sample performance) than cross-validation or repeated sampling (e.g., bootstrap methods) for most data sets. - Optimizing model tuning and selection on a singular test set produces an over specified model with poor generalization across unforeseen validation sets. - Specifically, a type of overfitting corrupts generalization estimates when model tuning and evaluation are performed on the exact same data set. - High variance in estimated model performance can also result from the particularities of the chosen test set (further amplified by case imbalance and small sample sizes). |
| K-fold CV^b^ | - Splits the entire dataset into *k* folds where every observation is assigned exactly once to a test fold containing 1/*k* of the entire dataset. The remaining observations are assigned to the training fold containing (*k*−1)/*k* of the original dataset (e.g., with *k*=5, each test fold contains 20% of the data and the training fold contains the remaining 80%). - Performed exhaustively ­ meaning every observation is assigned to exactly one test fold and contained within the training folds for every remaining split. - The estimated generalization performance is given by the average of the performance across all test folds. | - Straightforward evaluation method with ample documentation and educational resources, years of published research and performance results, and available open-source software packages. - Without incorporating extensive feature selection, hyperparameter tuning, and other model selection steps, the computational time required is entirely feasible for nearly all healthcare problems (especially with parallel computing). - In contrast to holdout validation, K-fold CV uses the entire data set for model development and evaluation. - Increasing the numbers of folds leads to higher variance and lower bias. Fewer folds lead to higher bias and lower variance. | - Theoretical results have shown that no unbiased estimator of the variance of K-Fold CV exists.^15^ - Model selection and parameter tuning must be performed independently. - For classification problems, standard (i.e., non-stratified) K-fold CV does not ensure equal proportions of cases between training and test folds. |
| LOOCV^c^ | - Leave one out cross-validation (LOOCV) is an extension of K-fold CV in which exactly one data point is held out as the test set while the remaining n−1 data points are used as the training set (equivalent to setting *k* as n−1 with n total data points). - This is also performed exhaustively such that each data point is used as the test set exactly once. | - Approximately unbiased estimate of true test error.^16^ | - Computational time required is intractable for most practical applications. - Discrimination metrics often become indeterminable with single records comprising each test set (e.g., calculating the AUROC^d^ for a binary classification problem). - Often associated with high variance.^17^ - LOOCV illustrates another instance of the BVT specific to cross-validation — as the number of folds increases the size of the training folds increase, leading to a less biased estimate of generalization performance (as each training fold becomes closer to representing the entire data set) while inducing higher variance due to smaller test sets leading to a large spread in test fold performance. |
| Stratified K-fold CV | - Stratified K-fold CV is performed identically to traditional K-fold CV with an additional criterion that every test and training split maintain equal proportions of cases (such that for binary classification problems the case prevalence is identical for train and test splits across each of the *k* folds). | - Reduces the bias and variance in estimated true test error for classification problems (required for extreme case imbalance). - Avoids model training failures due to insufficient minority class samples. | - Not applicable to regression problems with continuous outcomes. |
| Repeated K-fold CV | - Repeated K-fold CV is a procedure that simply involves re-running K-fold CV (optionally stratified K-fold CV as well) n times where the randomization of splitting into test and training folds ensures different sets of data are used for testing and training across repetitions of K-fold CV. - Estimated model performance is reported as the average of the evaluation metrics across repetitions of K-fold CV. | - Can provide more stable estimates that improve over K-fold CV (given the dependence of estimated model performance on the specific splits used within K-fold CV).^18,19^ - For most problems only 10 repeats are typically sufficient. | - Requires more computational time than K-fold CV (although parallel computing can mitigate increases in computational time). - Repeated K-fold CV can produce a wider range of reported performance metrics. |
| Nested CV | Nested cross-validation is a more complex procedure that involves specifying numbers of *outer* and *inner* folds to be used within two nested stages of K-fold CV **[Figure 1]**. First, K-fold CV is applied to the entire data set and for each of the *k* folds we do the following:   - Run an additional K-fold CV using only the outer training fold to produce inner train and inner test folds. - Perform all model selection steps (steps #2-5 in **[Textbox 1]**) to train models using different configurations of parameters and evaluate performance metrics on the inner test fold for each set of parameters. - Using the best set of parameters (based on a pre-specific metric), retrain a model on the entire data set used for the inner K-fold CV (the outer train split). - Generate predictions by applying this model to the outer test fold.   The estimated generalization performance is given by the average performance across all outer test folds. | - Provides a cross-validation routine for performing both model selection and model evaluation without increasing optimistic bias due to model parameters being tuned on the same data set that is used to report the estimated performance. | - More complicated to implement correctly, as doing so requires a sophisticated understanding of the differences between cross-validation for model evaluation and cross-validation for estimating the true out-of-sample prediction error. - Unavailable in many popular software libraries and typically requires developers to implement themselves. - Increased computational time required compared to non-nested cross-validation. |

^a^BVT: bias-variance tradeoff.

^b^CV: cross-validation.

^c^LOOCV: Leave One Out CV.

^d^AUROC: area under the receiver operator characteristic curve.
